# Depression, anxiety, and health related quality of life following radical prostatectomy and radiation treatment: 12 month outcomes in a South African cohort

**DOI:** 10.1186/s12894-026-02174-6

**Published:** 2026-06-12

**Authors:** Hayley Irusen, Pedro Fernandez, Tonya Esterhuizen, Andre van der Merwe, Pieter Spies, Soraya Seedat

**Affiliations:** 1 Division of Urology, Faculty of Medicine and Health Sciences, Stellenbosch University, Cape Town, South Africa; 2 Division of Epidemiology and Biostatistics, Department of Global Health, Faculty of Medicine and Health Sciences, Stellenbosch University, Cape Town, South Africa; 3 Department of Psychiatry, Faculty of Medicine and Health Sciences, Stellenbosch University, Cape Town, South Africa

**Keywords:** Depression, Anxiety, Health related quality of life (HRQOL), Prostate cancer, Prostatectomy, Radiation

## Abstract

**Purpose:**

Longitudinal patient-reported outcomes comparing radical prostatectomy (RP) and radiation (RT) for localized prostate cancer (CaP) in low- to middle- income countries are limited. We examined the comparative course of depression and anxiety and their association with health related quality of life (HRQOL) in men treated with RP or RT over a year post-treatment.

**Methods:**

Data from 161 South African (SA) men with CaP were analysed. Depression, Anxiety, HRQOL, and relevant covariates were measured at baseline, 3, 6, 9, and 12 months. We used the following validated scales: Centre for Epidemiologic Studies Depression (CES-D), State Trait Anxiety Inventory (STAI-S), European Organisation for Research and Treatment in Cancer Quality of Life (EORTC QLQ-PR25), Multidimensional Scale of Perceived Social Support (MSPSS), Memorial Anxiety Scale for Prostate Cancer (MAX-PC), Connor-Davidson Resilience Scale (CD-RISC), and Decisional Conflict Scale (DCS).

**Results:**

Depressive symptoms (CES-D ≥ 16) remained elevated 12 months post-RP (baseline: 43%; 3-month: 37%; 6-month: 47%; 12-month: 42%), while RT prevalence declined (39% to 29%). Similarly, anxiety (STAI-S ≥ 39) was more prevalent and persistent in the RP group (29% to 23%; 6-month peak: 28%) compared to the RT group (26% to 15%), where scores consistently declined throughout the first year. Overall urinary morbidity was marked in the first six months (β = 11.828; 95% CI: 7.931to 15.73; *p* < 0.001). More men in the RP group exhibited higher incontinence aids use (β = –21.477; 95% CI: to 33.261 to 9.694; p < 0.001). Sexual function in RP was lower (β = 18.006; 95% CI: 6.881 to 29.131; *p* = 0.002) with increased sexual activity from month nine onwards (β = –13.203; 95% CI: –23.489 to –2.916; *p* = 0.012). RT was associated with bowel symptoms in the latter half of follow-up. (4.681; 95% CI: 0.702 to 8.659; *p* = 0.021). Depression and anxiety adversely affected all HRQOL functional and symptom domains except sexual activity.

**Conclusion:**

The high prevalence of depression and anxiety reflects marked psychological morbidity in men treated for CaP in the first year post-treatment. Given the substantial association between psychological impairment and HRQOL, integrating mental health screening, management and referral into uro-oncology protocols is critical.

## Introduction

Ninety eight percent of men diagnosed with prostate cancer (CaP) have favourable ten year survival rates as a result of improved screening, diagnosis and treatments [1, 2]. However they are at high risk for depression and anxiety following diagnosis and treatment, including treatment-emergent symptoms such sexual and urinary side effects [3].

The prevalence of comorbid depression fluctuates across cancer types, with reported rates as high as 13% in lung cancer versus 6% in genitourinary malignancies [4]. Estimates suggest that co-morbid depressive disorders in oncological populations are 2 to 3 times greater than in the general population [5]. Brunckhorst et al. [6] reported a substantial psychiatric burden among men with CaP regardless of grade or stage, noting prevalence rates of 17.07% for depression, 16.86% for anxiety, and 9.85% for suicidality [6].

Several factors informed the study methodology. First, cognitive impairment frequently accompanies depression in older adults, affecting memory, processing speed, and executive function [7]. Cognitive impairments, can affect recall; [8] consequently, these were assessed at baseline to ensure the validity of patient-reported outcomes—addressing an existing gap in uro-oncology literature where cognitive pre-assessments to mitigate recall bias is frequently overlooked. Second, androgen deprivation therapy (ADT) prescribed for high-risk CaP can worsen mood, including depression and anxiety [9]. Finally, most research has focused on North American, European, and Oceanic samples, limiting generalisability to more diverse groups and therefore this study extends current literature by examining these effects within an underrepresented sample [10, 11].

While QOL covers all aspects of life, HRQOL specifically concerns the effects of health and medical interventions [12]. Consequently we characterise changes in symptoms of depression, anxiety and HRQOL in men receiving either RP or RT for localised CaP over time i.e., at baseline (when they were scheduled to come into hospital to receive curative treatments) and at twelve weekly time intervals over a year. We further evaluated whether there was a difference between men receiving RP and RT with respect to depression, anxiety and HRQOL over these intervals while assessing the effect of covariates such as CaP anxiety, resilience, social support and decisional conflict on depression, anxiety and HRQOL [13, 14]. Key variables such as resilience and social support, which significantly influence depression in CaP populations [15] including decisional conflict (DC), a factor associated with psychological well-being in patients making treatment-related decisions were also included [16].

### Study design and setting

This was a prospective observational study conducted at Tygerberg and Groote Schuur hospitals, two tertiary public health care facilities based in Cape Town, South Africa (SA), providing care to approximately 52 million South Africans, with 50 full time urologist nationwide [17].

#### Participants

Men with low- to favourable intermediate-risk CaP stratified according to the D’Amico Classification [18, 19] were invited to participate by the attending urologist. The D’Amico classification is a validated risk stratification tool for CaP, routinely incorporated into clinical practice guidelines by both the American and European Urological Associations. Medical staff involved with the management of patients were trained on the study protocol to assist with recruitment.

- Inclusion criteria: Men with low to favourable intermediate risk confirmed histologically and fulfilling the cognitive screening criteria.
- Exclusion criteria: Men treated with ADT or clinically diagnosed hypogonadism as per standard of care which excluded laboratory tests in the initial phase.

**Fig. 1:**
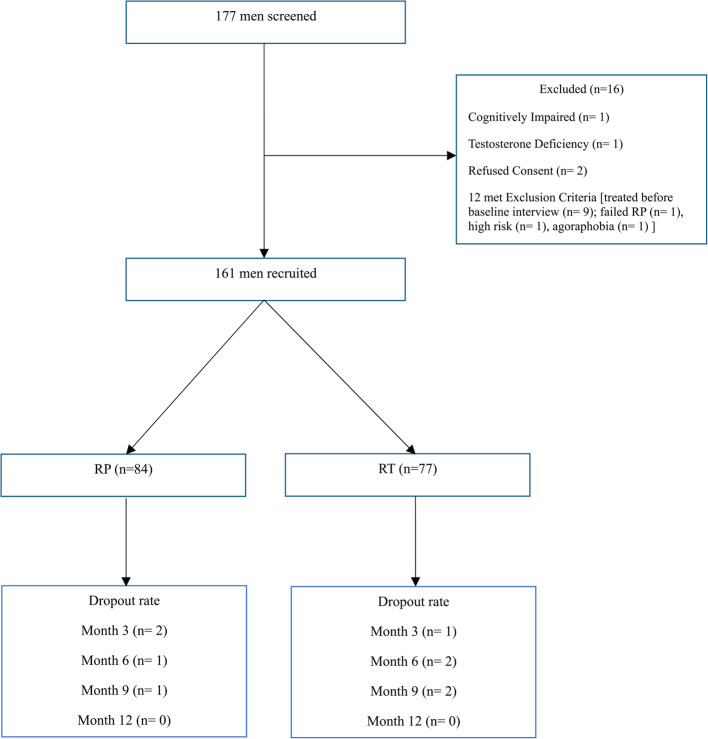
Flow diagram of study population. RP (Radical Prostatectomy), RT (Radiation Treatment)

A total of 177 men were screened of which 161 were included (Fig. 1 Flow Diagram). Data collection occurred between June 2019 to June 2023. Participants were pre-consented and underwent cognitive screening using a validated Six Item Screener (SIS) Questionnaire [20, 21] to reduce recall bias. Patients with two or fewer errors were invited to continue participation while those with more than two errors were referred to their local clinics for further management.

#### Interventions

Participants receiving either RP or RT were consecutively selected. Treatment decisions were based on clinical indications as determined by a multidisciplinary team, in accordance with international best practice [22].

#### Measures

Validated questionnaires included the Centre for Epidemiologic Studies Depression Scale (CES-D (≥ 16, depressive symptoms) and the State Trait Anxiety Inventory (STAI-S (≥ 39; ≥ 54, anxiety). Health-related quality of life was measured via the EORTC QLQ-PR25 (0–100 scale; higher scores denoting symptom severity or improved function). We also assessed CaP anxiety (MAX-PC), social support (MSPSS), resilience (CD-RISC), and decisional conflict (DCS) using previously described measures [13, 14, 23]. All questionnaires were translated ensuring linguistic and cultural appropriateness, and piloted.

#### Data collection

Baseline data were collected pre-treatment, concurrent with hospital admission for curative treatment (RP or RT), and re-administered at 12-week intervals for one year post-treatment. Study staff were on hand to assist patients when requested to ensure completeness and accuracy of questionnaires. All data was entered by a trained data clerk onto REDCaP, an electronic database with a secure server. Quality checks for correctness and missing data on REDCaP was done. Print and electronic data were de-identified by assigning a personal identification number (PIN) to each participant.

#### Statistical analysis

Power analysis in the study protocol was initially based on 110 participants per group, calculated using 80% power, minimum number of events per variable for longitudinal modelling of 10: 10 predictor variables and 10% attrition. However, the COVID-19 pandemic limited the sample size attainable. A post hoc power analysis was done for anxiety, depression, and HRQOL outcomes using the assumptions of the total sample size attained of 161 with equal sized groups, slope difference of 5.8 units with a standard deviation of 12 units, 5 time points and a 0.5 correlation between subsequent time points. Under these assumptions the power was 92.9%. Thus, these analyses were powered to detect effect sizes of 5.8 units or more between the two treatment groups. PASS (PASS 2021 Power Analysis and Sample Size Software (2021). NCSS, LLC, Kaysville, Utah, USA, ncss.com/software/pass) software was used for this power analysis.

Stata MP version 19 for windows (StataCorp. 2025. Stata 19. Statistical Software. College Station, TX: StataCorp LLC) was used in data analysis. Cronbach’s alpha coefficients were computed for items within scales to test internal consistency. Each outcome was separately analysed using linear mixed models with restricted maximum likelihood (REML). Independent variables modelled as fixed effects included 5 time points for all outcomes, treatment group (RT vs RP), interaction between time and treatment group, CES-D, STAI-S, MSPSS, MAX-PC, CD-RISC, DCS, age and heart disease. Age and heart disease were included as covariates because there appeared to be clinically important differences between treatment groups. Individual participant’s repeated observations were modelled as random effects. Model assumptions of normality of the outcome variable, homoscedasticity, and linearity were checked using standard diagnostic procedures, including inspection of residual plots and quantile – quantile plots for residuals and random effects. Variance inflation factors were computed for fixed effects models and found to indicate low multicollinearity (< 3) between fixed effects factors. Marginal plots of adjusted least squares means for time by treatment were plotted.

#### Ethical considerations

All participants provided written informed consent. Ethics approval was granted by Stellenbosch University (S19/01/019) and the University of Cape Town (HREC 418/2019). The study adhered to the Declaration of Helsinki, South African Good Clinical Practice and the South African Medical Research Council guidelines.

## Results

Table 1 shows the demographic and clinical characteristics of patients by treatment groups. Of the 161 men, 84 underwent RP and 77 received RT. Age was higher in the RT group (RP: 61.96 years, SD = 5.71; RT: 64.36 years, SD = 5.52). More men in the RT group reported use of cardiovascular medication compared to the RP group (31.2% vs. 15.5%).

**Table 1:** Demographic and clinical variables

	RP n = 84 (52%)	RT n = 77 (48%)	Total (n = 161)
Age in Years (mean, SD)	61.9 (5.71)	64.4 (5.52)	63.11 (5.73)
Race			
Black	7 (8.54)	12 (16.00)	19
Mixed Ancestry	66 (80.49)	52 (69.33)	118
Caucasian	9 (10.98)	11 (14.67)	20
Other	2 (1.2)	2 (1.2)	4
Education			
Primary School	16 (19.05)	18 (23.38)	34
High School	53 (63.10)	47 (61.04)	100
Tertiary Education	15 (17.86)	12 (15.58)	27
Employment status			
Formally Employed	17 (20.24)	18 (23.38)	35
Unemployed/Retrenched	12 (14.29)	12 (15.58)	24
Retired/Pensioner	43 (51.19)	40 (51.95)	83
Casual Worker	12 (14.29)	7 (9.09)	19
Marital Status			
Single (widowed, divorced, separated)	17 (20.24)	24 (31.17)	41
Married (in a relationship)	67 (79.76)	53 (68.83)	120
History of Smoking	71 (84.52)	61 (79.22)	132
History of Alcohol	76 (90.48)	65 (84.42)	141
Medical History			
Asthma	4 (4.76)	7 (9.09)	11
Heart Disease	13 (15.48)	24 (31.17)	37
High Blood Pressure	56 (66.67)	52 (67.53)	108
Diabetes	19 (22.62)	15 (19.48)	34
COPD	1 (1.19)	1 (1.30)	2
Kidney Disease	2 (2.38)	0.00	2
Peptic Ulcer	3 (3.57)	5 (6.49)	8
Arthritis	13 (15.48)	7 (9.09)	20

Cronbach’s alpha coefficients for the EORTC QLQ-PR25 indicated acceptable internal consistency for the urinary symptoms (0.85) and sexual functioning (0.71) domains, while the sexual activity domain was marginally below acceptable levels (0.66). This may be attributable to the limited number of items in this scale (two items), which can reduce internal consistency estimates. The coefficients for CES-D (0.77), STAI-S (0.92), CD-RISC (0.93), MAX-PC (0.94), MSPSS: family support (0.90), friends support (0.91), significant other support (0.93) indicated a relatively high internal consistency.

Among men undergoing RP, the prevalence of depressive symptoms (CES-D ≥ 16) was 43% at baseline, followed by 37%, 47%, 44%, and 42% at 3, 6, 9, and 12 months, respectively. Corresponding proportions in the RT sample were 39%, 29%, 31%, 31%, and 29% (Table 2). CES-D scores in the RT group were lower than RP particularly at month 9 (β = –1.839, 95% CI: –3.768 to 0.089; *p* = 0.062) as seen in Table 3. Increased values on the STAI-S (β = 0.286, 95% CI: 0.242 to 0.330; *p* < 0.001), MAX-PC (β = 0.161, 95% CI: 0.101 to 0.219; *p* < 0.001), and the DCS (β = 0.048, 95% CI: 0.015 to 0.082; *p* = 0.005) were associated with higher CES-D scores.

**Table 2:** Prevalence rates for depression and anxiety

	Baseline Treatment		3 months Treatment		6 months		9 months		12 months	
	RP	RT	RP	RT	RP	RT	RP	RT	RP	RT
	N (%)	N (%)	N (%)	N (%)	N (%)	N (%)	N (%)	N (%)	N (%)	N (%)
CESD	36 (42.9)	30 (39)	30 (36.6)	22 (28.9)	38 (46.9)	23 (31.1)	35 (43.8)	22 (30.6)	33 (41.3)	21 (29.2)
STAIS	24 (28.6)	20 (26)	20 (24.4)	14 (18.9)	23 (28.4)	15 (20.3)	15 (18.8)	10 (13.9)	18 (22.5)	11 (15.3)
STAIS2	2 (2.4)	2 (2.6)	5 (6.1)	1 (1.4)	7 (8.6)	2 (2.7)	6 (7.5)	2 (2.8)	6 (7.5)	0

**Table 3:** Linear Mixed Model—EORTC QLQ PR-25

	**Urinary Symptoms**		**Incontinence Aid**		**Bowel Symptoms**		**Sexual Activity**		**Sexual Functioning**		**CES-D**		**STAI-S**	
	**Coefficient (95% CI)**	***p*-value**	**Coefficient (95% CI)**	***p*-value**	**Coefficient (95% CI)**	***p*-value**	**Coefficient (95% CI)**	***p*-value**	**Coefficient (95% CI)**	***p*-value**	**Coefficient (95% CI)**	*p*-value	**Coefficient (95% CI)**	*p*-value
Treatment Group RT vs RP	−3.274 (−8.749—2.201)	0.241	2.972(−7.001—12.944)	0.559	−2.684 (−5.916—0.548)	0.104	−0.193 (−9.365—8.979)	0.967	4.022 (−5.092—13.137)	0.387	0.903 (−1.833—1.618)	0.903	−0.026 (−2.480—2.429)	0.984
Time 3 m vs baseline	11.828 (7.931—15.726)	< 0.001	18.997 (10.327—27.667)	< 0.001	0.287 (−2.438—3.012)	0.837	5.073 (−1.969—12.115)	0.158	−22.249 (−30.321—−14.178)	< 0.001	0.233 (−1.091—1.556)	0.730	−1.107 (−3.069 −0.856)	0.269
Time 6 m vs baseline	5.606 (1.692—9.519)	0.005	15.953 (7.035—24.871)	< 0.001	1.679 (−1.057—4.414)	0.229	6.724 (−0.346—13.794)	0.062	−29.919 (−37.788—−22.049)	< 0.001	0.643 (−0.685—1.971)	0.343	−1.519 (−3.489—0.449)	0.130
Time 9 m vs baseline	3.595 (−0.349—7.539)	0.074	12.251 (3.296—21.207)	0.007	−0.329 (−3.083—2.424)	0.815	9.887 (2.765—17.009)	0.007	−21.218 (−29.348—−13.089)	< 0.001	0.793 (−0.545—2.130)	0.245	−1.833 (−1.833—0.149)	0.070
Time 12 m vs baseline	−0.942 (−4.886—3.001)	0.640	11.433 (2.509—20.356)	0.012	−2.601 (−5.354—0.153)	0.064	8.449 (1.327—15.571)	0.020	−21.480 (−29.491—−13.469)	< 0.001	0.374 (−0.964—1.712)	0.584	−0.885 (−2.869—1.099)	0.382
Treatment Group * Time RT 3 months	−4.699 (−10.335—0.938)	0.102	−21.477 (−33.261—−9.694)	< 0.001	1.946 (−1.995—5.888)	0.333	0.991 (−9.194—11.175)	0.849	18.006 (6.881—29.131)	0.002	−1.312 (−3.224—0.600)	0.179	0.456 (−2.385—3.297)	0.753
treatment group*time RT 6 months	−4.328 (−9.979—1.323)	0.133	−19.159 (−31.239—−7.079)	0.002	0.900 (−3.049—4.850)	0.655	−5.011 (−15.242—5.221)	0.337	22.626 (11.865—33.388)	< 0.001	−1.146 (−3.063—0.772)	0.242	0.605 (−2.242—3.453)	0.677
treatment group * time RT 9 months	−3.510 (−9.205—2.185)	0.227	−16.688 (−28.774—−4.601)	0.007	4.681 (0.702—8.659)	0.021	−13.203 (−23.489—−2.916)	0.012	10.980 (0.102—21.858)	0.048	−1.839 (−3.768—0.089)	0.062	1.278 (−1.589—4.145)	0.382
treatment group * time RT 12 months	−0.3445 (−6.045—5.355)	0.906	−15.539 (−27.649—−3.430)	0.012	6.033 (2.051—10.015)	0.003	−11.509 (−21.804—−1.213)	0.028	16.518 (5.615—27.422)	0.003	−1.099 (−3.033- 0.834)	0.265	−0.245 (−3.116—2.627)	0.867
Age	−0.143 (−0.502—0.216)	0.436	−0.314 (−0.492—0.429)	0.894	−0.034 (−0.212—0.145)	0.712	1.633 (1.067—2.198)	< 0.001	−0.518 (−1.104—0.067)	0.083	0.076 (−0.031—0.182)	0.162	−0.099 (−0.244—0.045)	0.177
CESD	0.467 (0.254—0.679)	< 0.001	0.382 (0.005—0.759)	0.047	0.138 (0.001—0.276)	0.050	0.026 (−0.349—0.400)	0.894	−0.464 (−0.847—−0.081)	0.017	-	-	0.616 (0.523—0.709)	< 0.001
STAI-S	0.422 (0.277—0.007)	< 0.001	0.617 (0.368—0.867)	< 0.001	0.259 (0.164—0.354)	< 0.001	0.101 (−0.156—0.358)	0.441	−0.192 (−0.464—0.0792)	0.165	0.286 (0.242—0.330)	< 0.001	-	-
MSPSS	−0.465 (−1.823—0.893)	0.502	−1.966 (−4.181—0.249)	0.082	−1.085 (−1.924—−0.246)	0.011	0.922 (−1.427—3.270)	0.442	1.373 (−1.102—3.848)	0.277	−0.304 (−0.746—0.137)	0.176	−0.181 (−0.816—0.454)	0.576
MAX-PC	0.343 (0.158—0.528)	< 0.001	−0.071 (−0.387—0.245)	0.659	0.222 (0.107—0.337)	< 0.001	−0.092 (−0.412—0.229)	0.576	0.127 (−0.227—.480)	0.483	0.161 (0.101—0.219)	< 0.001	0.274 (−0.189—0.359)	< 0.001
CD-RISC	0.059 (−0.025—0.143)	0.168	0.084 (−0.057—0.225)	0.242	0.031 (−0.022—0.085)	0.251	0.014 (−0.132—0.161)	0.851	0.045 (−0.114—0.204)	0.578	0.009 (−0.018—0.038)	0.477	−0.158 (−0.196—−0.119)	< 0.001
DCS	0.079 (−0.023—0.182)	0.128	0.057 (−0.113—0.227)	0.510	0.038 (−0.027—0.103)	0.251	0.189 (0.011—0.368)	0.038	−0.046 (−0.250—0.158)	0.656	0.048 (0.015—0.082)	0.005	−0.015 (−0.064—0.033)	0.541
Heart Disease	−0.950 (−5.807—3.907)	0.701	4.512 (−1.987—11.011)	0.174	0.641 (−1.773—3.055)	0.603	5.479 (−2.165—13.123)	0.160	−1.684 (−9.859—6.491)	0.686	0.782 (−0.656—2.219)	0.287	−0.075 (−2.035—1.884)	0.940

In the RP group, the proportion of participants with STAI-S scores ≥ 39 was 29% at baseline, 24% at month 3, 28% at month 6, 19% at month 9, and 23% at month 12. In the RT group, the corresponding proportions were 26%, 19%, 20%, 14%, and 15%, respectively. Using the higher STAI-S threshold of ≥ 54, the prevalence in the RP group was 2.4% at baseline, increasing to 6.1% at month 3, 8.6% at month 6, and 7.5% at both months 9 and 12. By comparison, the RT group demonstrated rates of 2.6% at baseline, 1.4% at month 3, 2.7% at month 6, 2.8% at month 9 and 0% at month 12. No change in STAI-S scores was observed in the entire cohort over time nor between treatments, though a drop in scores was noted at month 9 (β = –1.833; 95% CI: –1.833 to 0.149; *p* = 0.070) as shown in Table 3. Higher CES-D scores (β = 0.616; 95% CI: 0.523 to 0.709; *p* < 0.001) and MAX-PC scores (β = 0.274; 95% CI: 0.189 to 0.359; *p* < 0.001) were associated with increased STAI-S scores. Conversely, lower CD-RISC scores (β = –0.158; 95% CI: –0.196 to –0.119; p < 0.001) were associated with increased STAI-S scores.

Based on patient-reported outcome measures (EORTC QLQ-PR25), urinary symptom scores increased for the total sample at month 3 (β = 11.828; 95% CI: 7.931 to 15.726; p < 0.001), month 6 (β = 5.606; 95% CI: 1.692 to 9.519; *p* = 0.005), and month 9, (β = 3.595; 95% CI: –0.349 to 7.538; p = 0.074) as seen in Table 3. RT urinary scores were lower than RP over the year with no evidence of a difference between treatments. Higher CES-D scores (β = 0.467; 95% CI: 0.254 to 0.679; *p* < 0.001), STAI-S scores (β = 0.422; 95% CI: 0.277 to 0.007; p < 0.001), and MAX-PC scores (β = 0.343; 95% CI: 0.158 to 0.528; *p* < 0.001) were associated with increased urinary symptom scores (Table 3).

Incontinence aid scores were lower among RT patients than RP patients at month 3 (β = –21.477; 95% CI: –33.261 to –9.694; p < 0.001), month 6 (β = –19.159; 95% CI: –31.239 to –7.079; *p* = 0.002), month 9 (β = –16.688; 95% CI: –28.774 to – 4.601; p = 0.007), and month 12 (β = –15.539; 95% CI: –27.649 to –3.430; p = 0.012) shown in Table 3. Higher CES-D (β = 0.382; 95% CI: 0.005 to 0.759; *p* = 0.047) and STAI-S scores (β = 0.617; 95% CI: 0.368 to 0.867; p < 0.001) were associated with increased incontinence aid scores.

Comparatively, bowel symptom scores were higher in RT group than RP group, particularly at 9 (β = 4.681; 95% CI: 0.702 to 8.659; *p* = 0.021) and 12 months (β = 6.033; 95% CI: 2.051 to 10.015; p = 0.003) as shown in Table 3. Higher CES-D (β = 0.138; 95% CI: 0.001 to 0.276; *p* = 0.050), STAI-S (β = 0.259; 95% CI: 0.164 to 0.354; *p* < 0.001), and MAX-PC scores (β = 0.222; 95% CI: 0.107 to 0.337; p < 0.001) were associated with increased bowel symptom scores. Conversely, lower MSPSS scores (β = –1.085; 95% CI: –1.924 to –0.246; *p* = 0.011) were associated with greater bowel symptom scores.

After adjusting for age, RT patients reported lower sexual activity in contrast to RP patients, particularly at 9 (β = –13.203; 95% CI: –23.489 to –2.916; *p* = 0.012) and 12 months (β = –11.509; 95% CI: –21.804 to –1.213; *p* = 0.028) as seen in Table 3. It should be noted that the Cronbach’s coefficient for sexual activity was low (0.66). Increased age (β = –1.633; 95% CI: 1.067 to 2.198; *p* < 0.001) and higher DSC scores (β = 0.189; 95% CI: 0.011 to 0.368; *p* = 0.038) were both associated with reduced sexual activity scores.

At all-time points, age adjusted sexual functioning scores were higher in the RT group compared with RP: month 3 (β = 18.006; 95% CI: 6.881 to 29.131; *p* < 0.002), month 6 (β = 22.626; 95% CI: 11.865 to 33.388; p < 0.001), month 9 (β = 10.980; 95% CI: 0.102 to 21.858; *p* = 0.048), and month 12 (β = 16.518; 95% CI: 5.615 to 27.422; *p* = 0.003) as noted in Table 3. Higher CES-D scores were associated with lower sexual functioning (β = –0.464; 95% CI: –0.847 to –0.081; *p* = 0.017).

## Discussion

While most studies examined depression, anxiety and HRQOL elsewhere in predominant North American, European and Oceanic samples not much is understood in the short term in African groups undergoing curative treatments at public health care facilities.

Treatment groups were similar demographically, except for age and cardiac medication use. Older men mainly received RT, whereas younger individuals underwent RP [24], which may also explain the higher usage of cardiac medication in the RT group [25].

While the general prevalence of depression and anxiety among South Africans 65 years and older was 6.5% and 7.2% [26], these figures were notably elevated in clinical settings. Specifically, psychological morbidity remained consistently high across both treatment groups, with up to half RP men and more than one-third of RT reporting depressive symptoms over time [25, 27]. A reduction in depressive symptoms was observed at nine months, which may be attributed to the corresponding decline in anxiety during the same period. Untreated mental health impairment has been shown to adversely affect treatment decisions, adherence to therapy, and increased mortality [28–30]. Concerningly, older adults (65 +) in SA, which includes most men with CaP, have the lowest odds of receiving treatment for depression and anxiety [31]. Depression was strongly associated with all domains of HRQOL with the exception of sexual activity [32].

Approximately one in five men receiving RP reported anxiety symptoms throughout the follow up, with a nine-month nadir (19%) whereas anxiety among RT men declined consistently (26% to 15%), with a marked reduction at nine months (14%). State anxiety and depression were strongly associated, suggesting comorbidity [33], which may partly be attributed to anxiety arising from the CaP diagnosis and treatments [34]. The strong inverse relationship between anxiety and resilience indicates that those with higher resilience may mitigate symptoms of both anxiety and depression [35].

Urinary symptoms substantially increased for the entire group at month three and six. Among men treated with surgery, urinary symptoms were comparatively worse, with increased use of incontinence aids throughout the follow-up, highlighting the severity of urinary morbidity. Prior research shows that RP is associated with urinary morbidity, with recovery extending beyond the first year following treatment [36]. The RP cohort reported comparatively higher sexual activity in the latter half of follow-up; however, even after age adjustment, sexual function remained markedly impaired despite continued engagement. Given the suboptimal internal consistency of the sexual activity domain, likely due to the limited number of items, these findings should be interpreted with caution. Substantial RP-related urinary and sexual morbidity highlights need for a multidisciplinary approach to surgical treatment. Public health facilities in SA must prioritize provision of sufficient incontinence support for at least one year following treatment.

Men receiving RT experienced worsening urinary symptoms, though to a lesser extent than RP, with a reduced need for incontinence aids, reflecting distinct treatment-related side-effect profiles. In contrast, bowel morbidity was more marked following RT, especially at nine and twelve months [11]. Even though sexual activity remained lower in the RT group after controlling for age, these men still reported substantially better sexual functioning than those undergoing RP. While RT may better preserve sexual function, this is counterbalanced by greater bowel morbidity, highlighting the importance of individualized discussions of treatment trade-offs.

Key strengths of this study include pre-enrolment cognitive screening, which improved the reliability of patient-reported data, and a prospective design that enabled longitudinal assessment of within-patient changes, thereby reducing recall bias and enhancing generalisability. Validated, culturally and linguistically adapted questionnaires and high internal consistency further support measurement reliability. Limitations include reduced sample size, one year follow-up post treatment, telephone interviews due to COVID-19, the latter potentially limiting visual cues, use of self-reported questionnaires that may overestimate symptoms, and exclusion of men with hypogonadism or on ADT, preventing assessment of their effects on depression, anxiety, and HRQOL.

Based on the findings of this study it can be concluded that post-treatment depression and anxiety remained highly prevalent and had a substantial effect on HRQOL irrespective of treatment received. Both treatments were associated with increased urinary morbidity within the first six months with greater severity in RP. While there was increased use of incontinence aids across the sample, dependence was considerably more pronounced in the RP group. Sexual activity increased in both treatment groups, but those in the RP group showed a substantially greater increase, accompanied by a more pronounced decline in sexual function. RT was linked to more severe bowel symptoms. Overall, depression and anxiety showed a marked association with HRQOL.

### Recommendations

Formalising structured mental health screening, management and appropriate referrals into uro-oncology protocols is critical to address the adverse effects of depression and anxiety on HRQOL. Utilizing the Patient Health Questionnaire (PHQ-9) [37], and mandatory clinician training in urology, will strengthen referral pathways. Furthermore, implementing the Depression Care for People with Cancer (DCPC) program offers a proven, cost-effective strategy to enhance psychological outcomes in uro-oncology at public health facilities [38]. These combined interventions are a clinical imperative for establishing a comprehensive, patient-centered standard of care in uro-oncology.

## Data Availability

All data generated or analysed during this study are included in this published article and its supplementary information files. Further data are available from the corresponding author on reasonable request.
